# An analysis of differences in Carbapenem-resistant *Enterobacterales* in different regions: a multicenter cross-sectional study

**DOI:** 10.1186/s12879-024-09005-9

**Published:** 2024-01-22

**Authors:** Bo Guo, Peili Li, Bingyu Qin, Shanmei Wang, Wenxiao Zhang, Yuan Shi, Jianxu Yang, Jingjing Niu, Shifeng Chen, Xiao Chen, Lin Cui, Qizhi Fu, Lin Guo, Zhe Hou, Hua Li, Xiaohui Li, Ruifang Liu, Xiaojun Liu, Zhengrong Mao, Xingguo Niu, Chao Qin, Xianrong Song, Rongqing Sun, Tongwen Sun, Daoxie Wang, Yong Wang, Lanjuan Xu, Xin Xu, Yuejie Yang, Baoquan Zhang, Dongmin Zhou, Zhaozhen Li, Yinyin Chen, Yue Jin, Juan Du, Huanzhang Shao

**Affiliations:** 1 Department of Critical Care Medicine, Henan Key Laboratory for Critical Care Medicine, Zhengzhou Key Laboratory for Critical Care Medicine, Henan Provincial People’s Hospital, People’s Hospital of Zhengzhou University, People’s Hospital of Henan University, Zhengzhou, Henan China; 2 Department of Public Utilities Development, Henan Provincial People’s Hospital, Zhengzhou, China; 3 Department of Microbiology Laboratory, Henan Provincial People’s Hospital, Zhengzhou, China; 4 Department of Critical Care Medicine, The Second People’s Hospital of Pingdingshan City, Pingdingshan, China; 5 Department of Critical Care Medicine, Nanyang Nanshi Hospital, Nanyang, China; 6 Department of Critical Care Medicine, Yellow River Central Hospital, Zhengzhou, China; 7 Department of Critical Care Medicine, The First Affiliated Hospital of Henan University of Science and Technology, Luoyang, China; 8 Department of Critical Care Medicine, The Seventh People’s Hospital of Zhengzhou, Zhengzhou, China; 9 Department of Critical Care Medicine, Zhengzhou Orthopedic Hospital, Zhengzhou, China; 10 Department of Critical Care Medicine, Henan Provincial Hospital of Traditional Chinese Medicine, Zhengzhou, China; 11 Department of Critical Care Medicine, Fuwai Central China Cardiovascular Hospital, Zhengzhou, China; 12 Department of Critical Care Medicine, The Third People’s Hospital of Henan Province, Zhengzhou, China; 13 Department of Critical Care Medicine, The Second Affiliated Hospital of Zhengzhou University, Zhengzhou, China; 14 Department of Critical Care Medicine, The First Affiliated Hospital of Henan University of Chinese Medicine, Zhengzhou, China; 15 Department of Critical Care Medicine, Zhengzhou People’s Hospital, Zhengzhou, 450000 China; 16 Department of Critical Care Medicine, The Fifth Affiliated Hospital of Zhengzhou University, Zhengzhou, China; 17 Department of Critical Care Medicine, Henan Provincial Chest Hospital, Zhengzhou, China; 18 Department of Critical Care Medicine, The First Affiliated Hospital of Zhengzhou University, Zhengzhou, China; 19 Department of Critical Care Medicine, The Third People’s Hospital of Zhengzhou, Zhengzhou, China; 20 Department of Critical Care Medicine, Huaihe Hospital of Henan University, Kaifeng, China; 21 Department of Critical Care Medicine, Zhengzhou Central Hospital, Zhengzhou, China; 22 Department of Critical Care Medicine, The First Affiliated Hospital of Henan University, Kaifeng, China; 23 Department of Critical Care Medicine, The Sixth People’s Hospital of Zhengzhou, Zhengzhou, China; 24 Department of Critical Care Medicine, The Third Affiliated Hospital of Xinxiang Medical College, Xinxiang, China; 25 Department of Critical Care Medicine, Henan Cancer Hospital, Zhengzhou, China

**Keywords:** Geographic location, Prevalence, Intensive care units, Bacterial, Drug resistance

## Abstract

**Objective:**

This study aimed to explore the characteristics of carbapenem-resistant *Enterobacterales* (CRE) patients in the intensive care unit (ICU) in different regions of Henan Province to provide evidence for the targeted prevention and treatment of CRE.

**Methods:**

This was a cross-sectional study. CRE screening was conducted in the ICUs of 78 hospitals in Henan Province, China, on March 10, 2021. The patients were divided into provincial capital hospitals and nonprovincial capital hospitals for comparative analysis.

**Results:**

This study involved 1009 patients in total, of whom 241 were CRE-positive patients, 92 were in the provincial capital hospital and 149 were in the nonprovincial capital hospital. Provincial capital hospitals had a higher rate of CRE positivity, and there was a significant difference in the rate of CRE positivity between the two groups. The body temperature; immunosuppressed state; transfer from the ICU to other hospitals; and use of enemas, arterial catheters, carbapenems, or tigecycline at the provincial capital hospital were greater than those at the nonprovincial capital hospital (*P* < 0.05). However, there was no significant difference in the distribution of carbapenemase strains or enzymes between the two groups.

**Conclusions:**

The detection rate of CRE was significantly greater in provincial capital hospitals than in nonprovincial capital hospitals. The source of the patients, invasive procedures, and use of advanced antibiotics may account for the differences. Carbapenem-resistant *Klebsiella pneumoniae* (CR-KPN) was the most prevalent strain. *Klebsiella pneumoniae carbapenemase* (KPC) was the predominant carbapenemase enzyme. The distributions of carbapenemase strains and enzymes were similar in different regions.

## Introduction

Carbapenem-resistant *Enterobacterales* (CRE) are widely disseminated in healthcare facilities worldwide and have a high mortality rate among patients due to the use of time-consuming detection methods and the limitations of treatment [1–4]. Compared to that of non-ICU isolates, the occurrence of CRE phenotypes was greater in the ICU [5]. In particular, rectal carbapenemase-producing Enterobacterales (CPE) colonization is highly prevalent in high-risk patients in the ICU [6]. Therefore, the timely and accurate detection of CRE, especially CPE, is crucial for infection prevention and clinical management, and early screening is the cornerstone of early detection. For CRE control, early detection of CRE acquisition with active surveillance may be useful [7]. Early detection and screening of CRE in patients in the ICU may promote early warning and reduce the risk of nosocomial CRE infections [8]. The use of admission screening for CRE and cohort care can significantly reduce the prevalence of CRE, the number of cases of hospital-acquired infections, and the length of hospitalization [9]. A study showed that 36.09% of CRE colonizations converted to infections. Therefore, CRE screening should be carried out in inpatients as early as possible, and effective intervention measures should be taken in a timely manner to avoid adverse consequences [10].

Pathogenic bacteria were distributed differently among different hospital departments and sample sources [11]. Thus, it is essential to target the prevention and control of key pathogenic bacteria in different hospital departments [11, 12]. However, the prevalence of CRE in different regions of Henan Province has not been reported. Zhengzhou is the most medically developed city in Henan Province, and the epidemiological characteristics of CRE may be distinctive. Therefore, to provide evidence for the targeted prevention and treatment of CRE, we screened 78 hospitals in different municipalities to determine the epidemiological characteristics of CRE in provincial and nonprovincial hospitals.

The population of Henan Province is nearly 100 million. CRE has been particularly prevalent in this area. To prevent and treat CRE in Henan Province, it is crucial to understand the characteristics of CRE in different regions.

## Materials and methods

### Research subjects

In this study, 1048 patients were enrolled from 96 ICUs in 78 hospitals in 18 cities. Patients aged older than 18 years were included. Patients with incomplete clinical data were excluded. All patients signed an informed consent form.

Of the 78 hospitals, 19 are provincial capital hospitals, all of which are tertiary hospitals, and 59 are nonprovincial capital hospitals, including 39 tertiary hospitals and 20 secondary hospitals (Table 1).

**Table 1:** Distribution of hospitals in different regions

	Provincial capital hospitals	Non-provincial capital hospitals
Tertiary hospital	19	39
Secondary hospital	0	20

Provincial capital hospital: The hospital is located in the provincial capital

### Study design and specimen collection

The trained ICU nurses collected one pharyngeal and two anal swabs per research subject on March 10, 2021. Microorganisms tested for one pharyngeal and anal swab specimen. For patients with CRE positivity, carbapenemase type was tested for in the other anal swab specimens. Following the guidelines of the People’s Republic of China Health Industry Standard and Specimen Collection and Transport in Clinical Microbiology (WS/T640-2018), samples were collected and transported. To derive samples, finish case reports, and follow up with patients, a doctor, and a nurse were assigned to each ICU.

The study was registered at http://www.chictr.org.cn/(registration number: ChiCTR2100044002, date of first registration: 06/03/2021) before patient enrollment.

### Bacterial isolation and identification

Isolation, culture and identification of bacteria were performed as previously reported [13].

### Identification of carbapenemase types

The most common carbapenemase types tested in all the CRE anal swabs included *Klebsiella pneumoniae* carbapenemase (KPC), New Delhi metallo-beta-lactamase (NDM), and oxacillinase-48 (OXA-48). The test was carried out as previously reported [13].

### Statistical analysis

KolmogorovThe Smirnov test was used to estimate the distribution of continuous variables. Normally distributed data were expressed as the means ± standard deviations and compared using t tests (*P* < 0.05). Nonnormally distributed data are expressed as medians (interquartile ranges) and were compared using the Mann–Whitney test (*P* < 0.05). Categorical variables are described as proportions and were compared using the chi-square test or Fisher’s test (*P* < 0.05). SPSS 26.0 was used for the statistical analysis, and a *P* value of < 0.05 was used to indicate statistical significance. The missing data were handled by multiple imputations [14] before analysis.

## Results

### Basic patient information

A total of 1009 patients were included in the study. Finally, 241 patients with positive CRE detection data were included in the analysis. The patients were divided into a provincial capital hospital group and a nonprovincial capital hospital group. There was no significant difference in age, sex ratio, or BMI between the two groups. See Table 2 for details.

**Table 2:** Comparison of basic information

	Provincial capital hospital (*n* = 92)	Non-provincial capital hospital (*n* = 149)	t/Z/χ2	*P*
**Age (years)**	60.97 ± 18.04	63.60 ± 16.85	-1.146	0.253
**Sex**				
Male	57	110	3.766	0.062
Female	35	39		
BMI	23.55 (20.82, 26.37)	23.44 (21.22, 25.83)	-0.726	0.469

*BMI* Body mass index

### CRE detection rate

The CRE detection rate was 23.89% in 96 ICUs of 78 hospitals in 18 cities, 28.22% in the provincial capital hospital and 21.82% in the nonprovincial capital hospital. There was a statistically significant difference between the groups (*P* = 0.027). See Table 3 for details.

**Table 3:** CRE detection rate in hospitals in different regions

	**Provincial capital hospital (326)**	**Non-provincial capital hospital (683)**	**χ2**	***P***
**Positive**	92 (28.22%)	149 (21.82%)	4.980	0.027
**Negative**	234 (71.78%)	534 (78.18%)		

### Laboratory tests and signs

The average body temperature was 37 °C in the Provincial Capital Hospital, which was significantly greater than that in the Nonprovincial Capital Hospital (*p =* 0.048). The average concentration of procalcitonin was 0.78, which was significantly greater than that in nonprovincial capital hospitals *(p* = 0.036). However, there were no significant differences in PaO2/FiO2, Acute Physiological and Chronic Health Evaluation Index II (APACHE II) score, white blood cell (WBC) count, C-reactive protein (CRP), creatinine, albumin, or total bilirubin. See Table 4 for details.

**Table 4:** Laboratory tests and signs

	Provincial capital hospital (*n* = 92)	Non-provincial capital hospital (*n* = 149)	t/Z/χ2	*P*
Body temperature (℃)	37.00 (36.60, 37.60)	36.80 (36.50, 37.20)	-1.981	0.048
PaO2/FiO2	264.50 (165.00, 375.75)	282.00 (205.50, 358.00)	-1.007	0.315
APACHE II score	18.00 (13.00, 22.00)	18.00 (13.00, 23.00)	-0.414	0.680
WBC (× 10^9^/L)	11.08 (7.70, 14.18)	10.44 (8.10, 13.91)	-0.174	0.862
CRP (mg/L)	52.85 (22.74, 110.40)	63.00 (23.68, 125.55)	-0.309	0.758
Procalcitonin (ng/ml)	0.78 (0.20, 2.25)	0.34 (0.12, 1.71)	-2.092	0.036
Creatinine (µmol/L)	59.00 (43.25, 103.50)	67.00 (42.42, 111.50)	-0.748	0.455
Albumin (g/L)	32.63 (28.00, 36.48)	32.00 (27.50, 36.05)	-0.336	0.738
Total bilirubin (µmol/L)	13.95 (9.23, 25.90)	12.70 (8.52, 23.90)	-0.686	-0.686

The APACHE II score was calculated from the lowest possible values in the first 24 hours after admission to the ICU, and the other parameters were collected on the day of sampling. *WBC* White blood cell, *CRP* C-reactive protein

### History of disease

Moreover, there was no statistically significant difference in the proportion of patients with comorbid diseases between the two groups. See Table 5 for details.

**Table 5:** Clinical data of patients at different regoin hospitals

	Provincial capital hospital (*n* = 92)	Non-provincial capital hospital (*n* = 149)	t/Z/χ2	*P*
**History of disease**				
Cardiovascular diseases	41 (44.57%)	73 (48.99%)	0.447	0.510
Chronic lung diseases	20 (21.74%)	18 (12.08%)	3.995	0.068
Chronic neurological diseases	19 (20.65%)	43 (28.86%)	2.005	0.174
Chronic kidney diseases	8 (8.70%)	10 (6.71%)	0.324	0.618
Peptic ulcer	4 (4.35%)	1 (0.67%)	2.191	0.072
Chronic liver diseases	4 (4.35%)	5 (3.36%)	0.002	0.735
Diabetes	15 (16.30%)	29 (19.46%)	0.380	0.609
HIV infection	2 (2.17%)	0	1.159	0.145
Connective tissue diseases	1 (1.09%)	1 (0.67%)	0	1.000
Blood diseases	0	2 (1.34%)	1.148	0.526
Malignant tumors	2 (2.17%)	9 (6.04%)	1.165	0.213
Immunosuppressed state	5 (5.43%)	0	5.811	0.008
**Source of patients**				
Newly admitted	28 (30.43%)	64 (42.95%)	20.874	< 0.001
Transferred from other departments of the same hospital	31 (33.70%)	63 (42.28%)		
Transferred from non-ICU departments of other hospitals	6 (6.52%)	11 (7.38%)		
Transferred from ICU of other hospitals	27 (29.35%)	11 (7.38%)		
Previous infection	78 (84.78%)	133 (89.26%)	1.047	0.321
Renal replacement therapy	16 (17.39%)	14 (9.40%)	3.336	0.074
Immunosuppressant used	6 (6.52%)	7 (4.70%)	0.099	0.567
**Intrusive or invasive procedures**	76 (82.61%)	139 (93.29%)	6.741	0.011
Invasive ventilator	50 (54.35%)	105 (70.47%)	6.442	0.013
Noninvasive ventilator	8 (8.70%)	14 (9.40%)	0.034	1.000
Surgery	33 (35.87%)	57 (38.26%)	0.138	0.784
Gastroscopy	6 (6.52%)	1 (0.67%)	4.985	0.013
Colonoscopy	0	1 (0.67%)	0	1.000
Enema	39 (42.39%)	28 (18.79%)	15.782	< 0.001
Central venous catheter	62 (67.39%)	107 (71.81%)	0.531	0.473
Arterial catheter	48 (52.17%)	37 (24.83%)	18.625	< 0.001
Gastric tube	63 (68.48%)	119 (79.87%)	3.990	0.064
Indwelling catheter	73 (79.35%)	133 (89.26%)	4.503	0.039
**Drainage tube**				
No drainage	59 (64.13%)	100 (67.11%)	10.691	0.038
Head	8 (8.70%)	29 (19.46%)		
Thoracic cavity	10 (10.87%)	9 (6.04%)		
Abdominal cavity	11 (11.96%)	7 (4.70%)		
Others	4 (4.35%)	4 (2.68%)		
**History of antibiotic exposure**				
Carbapenems	40 (43.48%)	45 (30.20%)	4.392	0.039
Betalactamaseinhibitors	41 (44.57%)	76 (51.01%)	0.945	0.355
3/4 generation Cephalosporin	7 (7.61%)	18 (12.08%)	1.223	0.288
Tigecycline	17 (18.48%)	5 (3.36%)	15.681	< 0.001
Polymyxins	3 (3.26%)	8 (5.37%)	0.197	0.539
Others	23 (25.0%)	35 (23.49%)	0.071	0.877

*HIV* Human immunodeficiency virus

### Patient sources

The proportion of patients transferred from ICUs to other hospitals in provincial capital hospitals was higher than that from nonprovincial capital hospitals. The proportion of patients who were newly admitted and transferred from other departments at the same hospital was greater than that from provincial capital hospitals. There was a statistically significant difference in the source of patients between the two groups (*p <* 0.001). See Table 5 for details.

### Intrusive or invasive procedures

The proportion of patients who underwent gastroscopy, enema or arterial catheterization at the provincial capital hospital was greater than that at the nonprovincial capital. However, there were more patients who received invasive ventilation at the nonprovincial capital hospital than at the provincial capital hospital. See Table 5 for details.

### History of antibiotic exposure

The proportion of patients using carbapenems and tigecycline in provincial capital hospitals was significantly greater than that in nonprovincial capital hospitals (*p* < 0.05). There was no significant difference in the use of other antibiotics between the two groups. See Table 5 for details.

### Patients’ length of hospital stay

The average length of hospital stay was 13.00 (6.00, 26.25) days for patients in provincial capital hospitals and 12.00 (6.00, 20.00) days for patients in nonprovincial capital hospitals. The average length of ICU stay was 10.50 (5.00, 23.00) days and 10.00 (5.00, 15.00) days in the two groups, respectively. There was no statistically significant difference in the length of hospital stay or ICU stay between the two groups. See Table 6 for details.

**Table 6:** Length of hospital stay

	Provincial capital hospital (*n* = 92)	Non-provincial capital hospital (*n* = 149)	t/Z/χ2	*P*
Length of hospital stay (days)	13.00 (6.00, 26.25)	12.00 (6.00, 20.00)	-0.793	0.429
Length of ICU stay (days)	10.50 (5.00, 23.00)	10.00 (5.00, 15.00)	-1.154	0.249

### Distribution of CRE strains

The study detected 346 cases of CRE in total, including 297 carbapenem-resistant *Klebsiella pneumoniae* (CR-KPN), 22 carbapenem-resistant *Escherichia coli* (CR-ECO), 6 carbapenem-resistant *Enterobacter cloacae* (CR-ECL), 19 CR-KPN/CR-ECO and 2 CR-KPN/CR-ECL strains. The isolation of CRE strains in the provincial capital hospital and in the nonprovincial capital hospital is shown in detail in Table 7. There was no statistically significant difference in the number of CRE strains detected between the two groups.

**Table 7:** Distribution of CRE strains

Strain	Provincial capital hospital	Non-provincial capital hospital	*P*
CR-KPN	118 (84.29%)	179 (86.89%)	0.553
CR-ECO	8 (5.71%)	14 (6.80%)	
CR-ECL	2 (1.43%)	4 (1.94%)	
CR-KPN/CR-ECO	11 (7.86%)	8 (3.88%)	
CR-KPN/CR-ECL	1 (0.71%)	1 (0.49%)	

The CR-KPN/CR-ECO samples contained two species: CR-KPN and CR-ECO. CR-KPN/CR-ECL samples contained two species: CR-KPN and CR-ECL

*CR-KPN* Carbapenem-resistant Klebsiella pneumoniae, *CR-ECO* Carbapenem-resistant Escherichia coli, *CR-ECL* Carbapenem-resistant Enterobacter cloacae

### Distribution of carbapenemase enzymes

Three carbapenemase enzymes were tested in 193 positive anal swabs, 150 KPC, 9 NDM, 11 KPC, and NDM, and 23 samples failed to contain the carbapenemase enzyme tested. The detection of carbapenemase enzymes in different regional hospitals was performed as follows (Table 8), and no significant differences were found between the two groups.

**Table 8:** Distribution of carbapenemase enzymes

Enzyme type	Provincial capital hospital	Non-provincial capital hospital	χ2	*P*
**KPC**	58 (75.32%)	92 (79.31%)	5.2	0.154
**NDM**	3 (3.90%)	6 (5.17%)		
**Both**	8 (10.39%)	3 (2.59%)		
**None**	8 (10.39%)	15 (12.93%)		

Both KPC and NDM were detected in one swab sample

None: Neither KPC nor NDM was detected in one swab

## Discussion

This was a multicenter cross-sectional study conducted in 96 ICUs of 78 hospitals in 18 cities; these included 19 provincial capital hospitals and 59 nonprovincial capital hospitals. Finally, 241 patients were analysed for differences in the clinical characteristics of CRE-positivity in different regions. The results revealed that the detection rate of CRE was 23.89%, 346 CRE-positive swabs were obtained, the proportion of CR-KPN was 85.84%, 193 anal swabs were tested for carbapenemase enzymes, and the proportion of KPC was 77.72%. The source of patients, invasive procedures, and history of antibiotic exposure may account for the higher detection rate of CRE in provincial capital hospitals.

Previous studies have shown that 22% of inpatients in the ICU had a subsequent CRE-positive swab [15], which is lower than the percentage in our study. The detection rate of CRE in hospitals in different regions varies. Our study showed that the detection rate of CRE was 28.22% in provincial capital hospitals and 21.82% in nonprovincial capital hospitals. According to our study, the high detection rate of CRE may be due to the source of the patients, invasive procedures, and history of antibiotic exposure. In this study, the proportion of patients transferred from ICUs to other hospitals in provincial capital hospitals was greater than that from nonprovincial capital hospitals. The patients were in critical condition and could not be handled by the local hospital. Previous studies have shown that the highest CRE positivity rate is observed in patients transferred from outside facilities to the ICU [16]. Our study showed that the proportion of patients who underwent gastroscopy, enema, or arterial catheter placement at the provincial capital hospital was greater than that at the nonprovincial capital. Interestingly, the number of patients who received invasive ventilation in nonprovincial capital hospitals was greater than that in the other hospitals. The proportion of patients using carbapenems and tigecycline in provincial capital hospitals was significantly greater than that in nonprovincial capital hospitals. Thus, CRE screening should be carried out in patients who are transferred from the ICUs of other hospitals, who are receiving invasive procedures and who are receiving advanced antibiotics as early as possible. In addition, standardized antibiotic use measures should be taken in a timely manner to avoid adverse consequences. It is effective to manage patients with CRE and non-CRE patients separately by isolating inpatients with CRE and dividing the population by the number of ICU staff [17]. Addressing sink traps can effectively reduce carbapenemase-producing Enterobacterales (CPE) transmission from patient to patient [18].

A previous study showed that a higher Sequential Organ Failure Assessment (SOFA) score, a nutritional risk in critically ill (NUTRIC) score, a prolonged intensive medical unit (ICU) length of stay (LOS), previous surgery, dialysis, mechanical ventilation during the ICU stay, and previous use of aminoglycoside and carbapenems were risk factors independently associated with CRE infection [14]. Gao et al. demonstrated that kidney disease, granulocytosis, invasive procedures, and CRE detection time were risk factors for CRE infection [10]. The invasive group had a greater rate of CRE infection than the noninvasive group [10]. Another study showed that urinary system disease, bronchoscopy, and combined antibiotic use were found to be independent risk factors for CPE colonization [6]. Otherwise, emergency department stays before ICU admission were associated with CRE colonization at admission to the ICU [19]. The length of hospital stay, hospital-acquired infection and treatment with carbapenem were found to be independent risk factors for CRE colonization [20]. A longer hospital stay was associated with an increased risk of CRE infection [21].

The study detected 346 cases of CRE in total, of which CR-KPN (85.84%) was the most prevalent strain. KPC (77.32%) is the major carbapenemase enzyme, and the detection rates of KPC and NDM were similar between the two groups. In addition, strains in which two carbapenemase enzymes were detected (KPC and NDM) were more prevalent in the provincial capital hospital. However, no significant differences were found between the two groups.

In a study by Han et al., 935 CRE strains were collected from 36 hospitals in 24 provinces or cities across China from 2016 to 2018. Overall, carbapenemases, including KPC-2 (51.6%), NDM (35.7%), and OXA-48-like carbapenemases (7.3%), were produced in 97.4% of the CRE strains [22].

Lin’s study collected 128 unique CRE isolates from 128 patients in the ICU of Chongqing, China: 69 (53.9%) CPE patients and 59 (46.1%) non-CPE patients. The most prevalent CPE isolates were bla (KPC-2) (56.5%), bla (NDM) (39.1%), and bla (IMP) (5.8%) [23].

Patients with CRE that produce carbapenemase were more likely to develop CRE infections than were those with non-carbapenemase-producing CRE [15]. KPC-CRE-colonized patients were particularly susceptible to subsequent CRE infections during their hospital stay [24]. Therefore, special attention should be given to patients with CREs harboring KPC enzymes.

Our study preliminarily confirmed the epidemiological characteristics of CRE at different hospital levels and provided a reference for the treatment of CRE locally. The limitations of our study include the small sample size and cross-sectional nature. This study did not explore the proportion of colonized CRE strains that converted to infection or distinguish CRE colonization from infection. Moreover, only common carbapenemase enzymes were tested for analysis due to the limitations of the finances. We will expand the sample size and optimize the experimental design to further validate our findings in the future.

## Conclusions

The detection rate of CRE was significantly greater in the provincial capital hospitals than in the nonprovincial capital hospitals. The source of the patients, invasive procedures, and the use of advanced antibiotics may account for the differences. CR-KPN was the most prevalent strain. KPC was the predominant carbapenemase enzyme. The distributions of carbapenemase strains and enzymes were similar in different regions.

## Data Availability

The datasets generated from the current study are available from the corresponding author upon reasonable request.
